# Assessing evidence for adaptive evolution in two hearing-related genes important for high-frequency hearing in echolocating mammals

**DOI:** 10.1093/g3journal/jkab069

**Published:** 2021-01-30

**Authors:** Hui Wang, Hanbo Zhao, Yujia Chu, Jiang Feng, Keping Sun

**Affiliations:** 1 College of Life Science, Jilin Agricultural University, Changchun 130118, China; 2 Jilin Provincial Key Laboratory of Animal Resource Conservation and Utilization, Northeast Normal University, Changchun 130117, China

**Keywords:** adaptive echolocation, bats, echolocation, high-frequency hearing

## Abstract

High-frequency hearing is particularly important for echolocating bats and toothed whales. Previously, studies of the hearing-related genes *Prestin*, *KCNQ4*, and *TMC1* documented that adaptive evolution of high-frequency hearing has taken place in echolocating bats and toothed whales. In this study, we present two additional candidate hearing-related genes, *Shh* and *SK2*, that may also have contributed to the evolution of echolocation in mammals. *Shh* is a member of the vertebrate Hedgehog gene family and is required in the specification of the mammalian cochlea. *SK2* is expressed in both inner and outer hair cells, and it plays an important role in the auditory system. The coding region sequences of *Shh* and *SK2* were obtained from a wide range of mammals with and without echolocating ability. The topologies of phylogenetic trees constructed using *Shh* and *SK2* were different; however, multiple molecular evolutionary analyses showed that those two genes experienced different selective pressures in echolocating bats and toothed whales compared to nonecholocating mammals. In addition, several nominally significant positively selected sites were detected in the nonfunctional domain of the *SK2* gene, indicating that different selective pressures were acting on different parts of the *SK2* gene. This study has expanded our knowledge of the adaptive evolution of high-frequency hearing in echolocating mammals.

## Introduction

Echolocation is a complex sensory system, usually used for orientation and feeding in environments where visibility is limited (Speakman 1993; Arch and Narins 2008). Echolocation has evolved independently in bats and whales in a remarkable case of adaptive phenotypic convergence driven by natural selection (Li *et al.* 2010; Liu *et al.* 2012; Shen *et al.* 2012). High-frequency hearing is an important component of echolocation and is essential for echolocators to perceive ultrasonic signals (Madsen *et al.* 2004; Li *et al.* 2008; Churchill *et al.* 2016). The molecular bases underlying echolocation accompanied by high-frequency hearing have attracted increasing attention.

Previously, several hearing-related genes have been reported to be related to the evolution of high-frequency hearing in both echolocating bats and toothed whales. *Prestin* is one of the most well-known hearing-related genes that has undergone convergent molecular adaptation for high-frequency hearing in echolocating bats and toothed whales (Li *et al.* 2008, 2010). Similarly, other hearing-related genes, including *KCNQ4*, *TMC1*, and P*jvk*, have also been reported to have undergone adaptive convergent or parallel evolution in echolocating mammals (Liu *et al.* 2011, 2012; Davies *et al.* 2012; Shen *et al.* 2012). Furthermore, comparative genomic analyses were conducted to uncover the genetic bases for high-frequency hearing in various echolocators, and these studies have provided a large number of candidate genes involved in echolocation and ultrasonic hearing (Thomas and Hahn 2015; Liu *et al.* 2018; Wang *et al.* 2020). Nevertheless, it is still necessary to conduct detailed and careful molecular evolutionary analyses of the candidate hearing-related genes in echolocating mammals (Parker *et al.* 2013; Zou and Zhang 2015; Wang *et al.* 2020).

Sonic hedgehog (*Shh*) is one of the three genes in the vertebrate Hedgehog gene family (Pereira *et al.* 2014); the gene plays an important role in the development of the inner ear. During inner ear development, *Shh* secreted from the notochord and floor plate is necessary for the specification of ventral otic fates and the mammalian cochlea (Riccomagno *et al.* 2002, 2005). In *Shh*-lacking mouse embryos, dorsoventral positioning within the otic vesicle is disrupted, and the cochlear duct and saccule fail to form (Brown and Epstein 2011). *Shh* is also involved in the cell fate determination of stato-acoustic ganglion neurons and in hair cell development in the inner ear (Fantetti and Fekete 2012; Groves and Fekete 2012). Moreover, *Shh* has been demonstrated to play an extrinsic role in mediating tonotopic organization of the mammalian organ of Corti (Son *et al.* 2015). Altogether, considering the important roles of *Shh* in the auditory system and the high-frequency hearing sensitivity of echolocating bats and toothed whales, we suggest that *Shh* may be involved in the adaptive evolution of echolocating bats and toothed whales.

The small-conductance Ca^2+^-activated K^+^ (SK) current is expressed in auditory hair cells of various vertebrates (Tucker and Fettiplace 1996; Yuhas and Fuchs 1999; Oliver *et al.* 2000; Marcotti *et al.* 2004). There are three genes, *SK1*, *SK2*, and *SK3*, encoding SK channels in the mammalian brain; however, only SK2 type channels are present in the cochlea (Nie *et al.* 2004). The *SK2* gene is expressed in both inner and outer hair cells of the mammalian cochlea (He and Dallos 1999; Marcotti *et al.* 2004). Previous studies demonstrated that SK2 is important in the SK channel contribution to excitatory postsynaptic potentials and directed synaptic localization (Johnson *et al.* 2007; Allen *et al.* 2011). Therefore, we suggest that the *SK2* gene may associated with the adaptive evolution of high-frequency hearing in echolocating mammals.

In this study, we applied comprehensive analyses to uncover the molecular adaptations of *Shh* and *SK2* in the evolution of high-frequency hearing in echolocating bats and toothed whales. For both tested genes, a wide range of mammals with or without echolocating ability were collected and sequenced to detect whether either the gene shows evidence of convergence/parallel evolution and molecular adaptation associated with the evolution of high-frequency hearing. This study is expected to provide new evidence concerning the genetic basis underlying the adaptive evolution of high-frequency hearing in echolocating mammals.

## Materials and methods

### Taxonomic coverage

We obtained 29 *Shh* coding region sequences for echolocating mammals (17 species from 8 families) and nonecholocating mammals (12 species from 8 families) (Table 1). In detail, 18 *Shh* coding sequences for mammals with or without echolocating ability were obtained by searching the NCBI database (www.ncbi.nlm.nih.gov). The species comprised two frequency-modulated (FM) bats (*Myotis davidii* and *Eptesicus fuscus*), two nonecholocating bats (*Pteropus vampyrus* and *Pteropus alecto)*, four echolocating toothed whales (*Tursiops truncatus*, *Orcinus orca*, *Lipotes vexillifer*, and *Physeter catodon*), two nonecholocating baleen whales (*Balaenoptera acutorostrata* and *Balaenoptera bonaerensis*), and eight other nonecholocating mammals (*e.g., Homo sapiens* and *Gorilla gorilla*). In addition, for a wider coverage of bat species, we sampled and sequenced five constant-frequency (CF) bats (*Rhinolophus ferrumequinum*, *Rhinolophus luctus*, *Hipposideros armiger*, *Hipposideros larvatus*, and *Hipposideros pratti*) and six other FM bats (*Myotis ricketti*, *Pipistrellus abramus*, *Nyctalus plancyi*, *Plecotus auritus*, *Tadarida teniotis*, and *Taphozous melanopogon*).

**Table 1:** Basic information from 29 mammals employed for the *Shh* gene

Order	Family	Species	Sequence source
Chiroptera	Rhinolophidae	***Rhinolophus ferrumequinum***	**KX495649**
		***Rhinolophus luctus***	**KX495650**
	Hipposideridae	***Hipposideros armiger***	**KX495651**
		***Hipposideros larvatus***	**KX495652**
		***Hipposideros pratti***	**KX495653**
	Pteropodidae	*Pteropus vampyrus*	XM_011370398.1
		*Pteropus alecto*	XM_006904474.1
	Vespertilionidae	*Myotis davidii*	XM_006767162.1
		***Myotis ricketti***	**KX495654**
		***Pipistrellus abramus***	**KX495655**
		***Nyctalus plancyi***	**KX495656**
		*Eptesicus fuscus*	XM_008156444.1
		***Plecotus auratus***	**KX495657**
	Molossidae	***Tadarida teniotis***	**KX495658**
	Emballonuridae	***Taphozous melanopogon***	**KX495659**
Cetacea	Balaenopteridae	*Balaenoptera acutorostrata*	XM_007165489.1
		*Balaenoptera bonaerensis*	BAUQ01160701.1
	Delphinidae	*Tursiops truncates*	XM_004311374.1
		*Orcinus orca*	XM_004281230.1
	Lipotidae	*Lipotes vexillifer*	XM_007457176.1
	Physeteridae	*Physeter catodon*	XM_007124669.1
Artiodactyla	Bovidae	*Bos mutus*	XM_005902017
Artiodactyla	Suidae	*Sus scrofa*	NM_001244513.1
Perissodactyla	Equus caballus	*Equus caballus*	XM_001914885.1
Rodentia	Muridae	*Mus musculus*	NM_009170.3
		*Rattus norvegicus*	NM_017221.1
Primate	Hominidae	*Homo sapiens*	NM_000193.3
		*Gorilla gorilla*	XM_004046549.1
	Callitrichidae	*Callithrix jacchus*	XM_002807047.3

Note: Bat species sequenced in this study are listed in bold.

A total of 27 *SK2* coding region sequences were obtained for mammals with or without echolocating ability (Table 2). In detail, 22 *SK2* coding region sequences were collected by searching NCBI, including one CF bat (*Rhinolophus sinicus*), four FM bats (*Myotis lucifugus*, *E. fuscus*, *Desmodus rotundus*, and *Miniopterus natalensis*), one click bat (*Rousettus aegyptiacus*), two nonecholocating bats (*P. vampyrus* and *P. alecto*), six echolocating toothed whales (*T. truncatus*, *O. orca*, *L. vexillifer*, *P. catodon*, *Neophocaena asiaeorientalis*, and *Delphinapterus leucas*), one nonecholocating baleen whale (*B. acutorostrata*), and seven other nonecholocating mammals (*e.g., Homo sapiens* and *Gorilla gorilla*). For a wide coverage of echolocating mammals, another five bat species were also sampled and sequenced, three CF bats (*R. ferrumequinum*, *H. armiger*, and *H. larvatus*), one FM bat (*M. ricketti*), and one-click bat (*Rousettus leschenaultii*).

**Table 2:** Basic information from 27 mammals employed for the *SK2* gene

Order	Family	Species	Sequence source
Chiroptera	Rhinolophidae	***Rhinolophus ferrumequinum***	**MT822706**
		*Rhinolophus sinicus*	XM_019727805.1
	Hipposideridae	***Hipposideros armiger***	**MT822707**
		***Hipposideros larvatus***	**MT822708**
	Vespertilionidae	***Myotis ricketti***	**MT822709**
		*Eptesicus fuscus*	XM_008144465.1
		*Myotis lucifugus*	XM_014462066.2
	Miniopteridae	*Miniopterus natalensis*	XM_016224945.1
	Phyllostomidae	*Desmodus rotundus*	XM_024563297.1
	Pteropodidae	***Rousettus leschenaulti***	**MT822710**
		*Rousettus aegyptiacus*	XM_016147342.1
		*Pteropus vampyrus*	XM_011384549.1
		*Pteropus alecto*	XM_006913288.1
Cetacea	Balaenopteridae	*Balaenoptera acutorostrata*	XM_007192113.1
	Delphinidae	*Tursiops truncatus*	XM_019940842.1
		*Delphinapterus leucas*	XM_022590999.1
		*Orcinus orca*	XM_004267462.2
	Lipotidae	*Lipotes vexillifer*	XM_007451206.1
	Phocoenidae	*Neophocaena asiaeorientalis*	XM_024743366.1
		*Physeter catodon*	XM_024122151.1
Perissodactyla	Equidae	*Equus caballus*	XM_023617854.1
Artiodactyla	Bovidae	*Bos Taurus*	XM_024997305.1
Primates	Cercopithecidae	*Macaca nemestrina*	XM_011772881.2
	Hominidae	*Pan troglodytes*	XM_016953646.2
		*Homo sapiens*	XM_011543389.1
Rodentia	Muridae	*Mus musculus*	NM_001312905.1
		*Rattus norvegicus*	XM_006254683.3

Note: Bat species sequenced in this study are listed in bold.

For wild-sampled bat species, a small piece of the wing membrane was biopsied. Thereafter, the bats were freed as soon as possible. The sample collection procedures followed the ethical principles of the National Animal Research Authority of Northeast Normal University, China (approval number: Nenu-20080416) and the Forestry Bureau of Jilin Province of China (approval number: [2006]178).

Genomic DNA was extracted from bat wing membrane biopsy samples using a UNIQ-10 column animal genomic DNA isolation kit (Sangon, Shanghai, China). Primers (Supplementary Table S1) for the *Shh* gene were designed according to the homologous sequence of *R. ferrumequinum* (AWHA01046314.1) from NCBI and Microbat (ENSMLUG00000025004.1) from Esembl (www.ensembl.org). Primers (Supplementary Table S1) for the *SK2* gene were designed according to the homologous sequences of *R. ferrumequinum* (AWHA01185014.1), *M. lucifugus* (AAPE02000266.1), and *P. vampyrus* (ABRP02162960.1) from NCBI. All primers were designed using Primer Premier 6 and evaluated by Oligo 8, and then synthesized by Sangon Biotech (Shanghai, China). Primers designed in this study may not be unique in all the newly sequenced bat species (Supplementary Table S1); therefore, redesign or reassessment of these primers will be necessary according to the specific bat species.

Polymerase chain reactions (PCRs) were performed in 50 µl volumes containing 25 µl of mix (Tiangen, Beijing), 2 µl of each primer (10 pmol/µl), 2 µl genomic DNA (10–100 ng), and 19 µl ddH_2_O. Cycling parameters were as follows: 94 °C for 5 minutes; 45 cycles at 94 °C for 30 s, Tm (Supplementary Table S1) for 30 s, and 72 °C for 1 minute; and a final extension at 72 °C for 10 minutes. All PCR products were isolated from a 1% agarose gel and cloned into the T-vector (TaKaRa). For cloning, positive clones were sequenced in both directions on an ABI 3730 Sequencer (Applied Biosystems).

### Phylogenetic Reconstruction

Separately, the coding region sequences of *Shh* and *SK2* for all available mammals were aligned using CLUSTAL W (Thompson *et al.* 1994) and MUSCLE 3.8.31 (Edgar 2004). Two methods were used to reconstruct phylogenetic trees, including Bayesian inference (BI) and maximum likelihood (ML). For BI trees, the best-fit model was selected by jModeltest (Posada 2008) according to Bayesian information criteria (BIC), and then HKY+G and TrN+I + G were selected for *Shh* and *SK2*, respectively. Subsequently, Bayesian phylogenetic reconstruction was conducted using MrBayes 3.2.0 (Huelsenbeck and Ronquist 2001; Ronquist *et al.* 2012). A Markov Chain Monte Carlo (MCMC) run with four simultaneous chains and 10 million generations was set, including a burn-in step corresponding to the first 2.5 million generations. ML trees were reconstructed using RAxML 7.0.4 (Stamatakis 2008). T92+G and T92+G + I with 10,000 bootstrap replicates were selected as the best models for the phylogenetic reconstruction of *Shh* and *SK2*, respectively.

### Molecular evolution analyses

To explore the heterogeneous selection pressures acting on both echolocating and nonecholocating mammals, sliding window analyses were performed for *Shh* and *SK2* using the program SWAAP 1.0.2 (Pride 2000). We estimated the nonsynonymous (dN) and synonymous (dS) substitution rates (the dN/dS ratio, termed omega ω) according to the Nei and Gojobori method (Nei and Gojobori 1986). Window size and step size were set to 90 and 9 bp, respectively. Higher ω values in echolocating mammals could be due to stronger selective pressures or lower selective constraints relative to nonecholocating mammals; moreover, lower selective pressures or higher selective constraints in nonecholocating mammals could also lead to higher ω values in echolocating mammals. Observing relatively high estimates of ω values in echolocating mammals may suggest important evolutionary implications, especially in light of the distinctive biology of high-frequency hearing in echolocating mammals. Subsequently, for both *Shh* and *SK2*, the ω values detected in echolocating mammals were compared with those detected in nonecholocating mammals, and the significance of differences between two groups of ω values were tested by an independent-sample *t*-test at a significance level of *P *<* *0.05 using SPSS (Arbuckle 2010).

**Figure 1.**
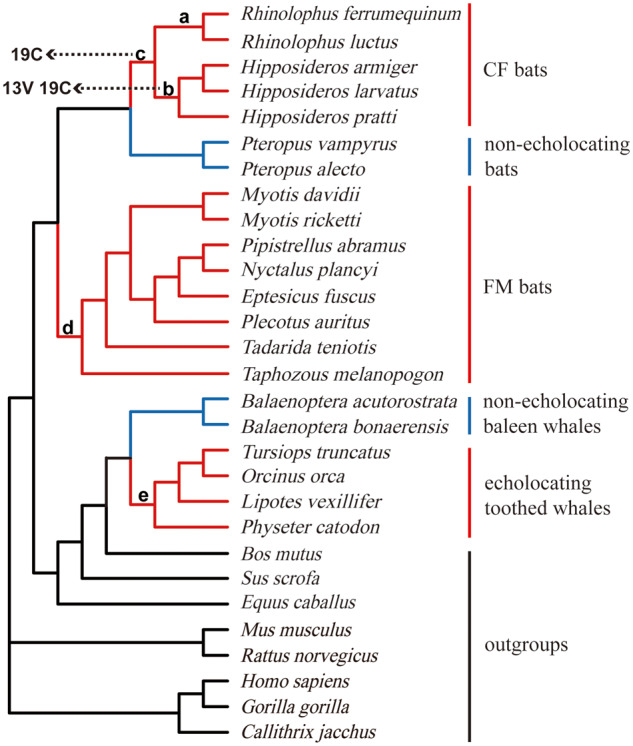
A well-established species tree of 29 mammals based on previous studies, here employed for the *Shh* gene analyses. Branch colors are as follows: echolocating bats and toothed whales (red), nonecholocating bats and baleen whales (blue), other nonecholocating mammals (black). Letters from a–e indicate foreground branches to be tested for the Branch model and the Branch-site model. Amino acid substitutions with ω > 1 are shown.

**Figure 2.**
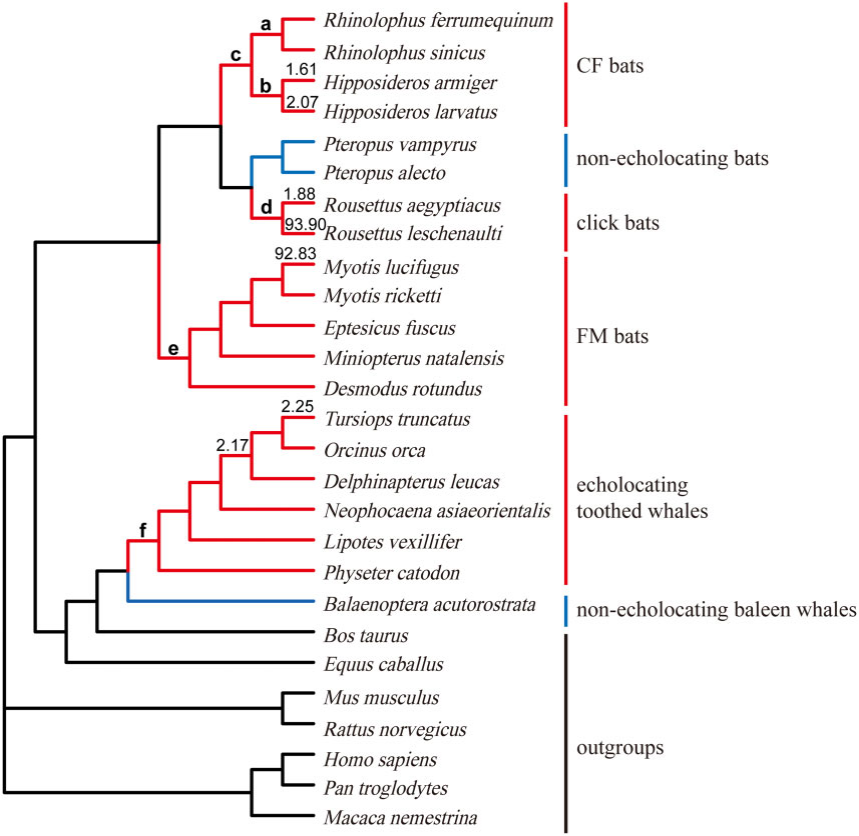
A well-established species tree of 27 mammals based on previous studies, here employed for the *SK2* gene analyses. Branch colors are as follows: echolocating bats and toothed whales (red), nonecholocating bats and baleen whales (blue), other nonecholocating mammals (black). Letters from a–f indicate foreground branches to be tested for the Branch model and the Branch-site model. Seven branches with ω > 1 were labeled with corresponding numbers.

The selective pressures in echolocating bats and toothed whales were further estimated for each gene using different codon substitution site models implemented in PAML 4.8 (Yang 2007). By comparing ω among sites and branches, the form and intensity of natural selection can be revealed, with ω < 1, ω = 1, and ω > 1 indicating negative selection, neutral evolution, and positive selection, respectively. Well-established species trees based on previously reported phylogenetic studies were used for *Shh* (Figure 1) and *SK2* (Figure 2) (Murphy 2001; Giannini and Simmons 2003; Hoofer and Bussche 2003; Jones and Teeling 2006; Au and Simmons 2007; Gatesy *et al.* 2013). In addition, we repeated the selection tests based on the putative gene trees topologies for *Shh* and *SK2*, respectively.

In detail, the Site model, the Branch model, and the Branch-site model with paired alternative hypothesis and null hypothesis were all performed to identify positively selected signals in targeted branches of each gene. Targeted branches (including each separate echolocating species and various combined groups of echolocating bats and toothed whales) were set as the foreground branch in turn for *Shh* and *SK2*. A likelihood ratio test was established to compare a model that allows particular sites to be under positive selection (ω > 1) on the foreground branch with a null model in which sites may evolve neutrally (ω = 1) or under purifying selection (ω  <  1) with an adjusted *P*-value <0.05 (Yang 2007).

For the Branch model, we first compared the free-ratio model with the one-ratio (M0) model. The free-ratio model assumes that there are different ω values in different branches, whereas the M0 assumes that ω is the same across all branches. We then compared the two-ratio model with the M0 to estimate the selective pressure of foreground branches. The two-ratio model assumes that foreground branches have a different ω value than background branches. All tested foreground branches (except for each separate echolocating species) are marked using lowercase in Figures 1 and 2. The statistic 2*ΔL* (twice the log likelihood difference between the nested models) was compared with the chi-square distribution (d.f. = 1, at a critical level of 0.05). To be more reasonable and rigorous, we performed a RELAX Branch model implemented in Datamonkey (http://www.datamonkey.org/) to detect selective pressures for all tested foreground branches.

For the Site model, M0, M1a, M2a, M3, M7, and M8 were included. Three pairwise comparisons of alternative hypothesis vs. null hypothesis, M1a vs. M2a, M7 vs. M8, and M0 vs. M3 were performed to detect positively selected sites for each gene. In addition, five other models implemented in Datamonkey [SLAC (single-likelihood ancestor counting), FEL (fixed-effects likelihood), REL (random-effects method), MEME (mixed-effects model of evolution), and FUBAR (fast unbiased Bayesian approximation)] were also performed to detect potential positively selected sites.

To conduct the in-depth adaptive evolutionary analysis, the Branch-site model was used to test for evidence of positive selection acting at sites along foreground branches. Specifically, under Model A, the ω values were assigned to four predefined site classes: 0 < ω_0_ < 1, ω_1_ = 1, ω_2a_ (could exceed 1 on the foreground but is constrained to be under purifying selection on the background) and ω_2b_ (could exceed 1 on the foreground but not on the background). Model A was compared with the null Model A where ω_2a_ = 1 using LRT with d.f. = 1 at a critical level of 0.05. The comparison between these two models is called test 2. In addition, test 1 was also used for the analyses, here comparing model A with M1a (neutral), with d.f. = 2. If Model A was a better fit, a posterior probability greater than 0.95 based on the Bayes Empirical Bayes (BEB) results was used to identify positively selected sites.

Moreover, given the different model comparisons that we have performed on these two genes, multiple test correction was performed using the Benjamini-Hochberg method based on an FDR adjusted *P*-value <0.05.

### Identification of parallel/convergent sites among echolocating mammals

To determine whether similar evolutionary patterns have occurred in echolocating bats and toothed whales that have independently developed the ability to echolocate and are habitually exposed to high-frequency sound but live in diverse environments, we searched for parallel/convergent amino acid substitutions from the internal nodes to terminal branches along paraphyletic lineages of echolocating mammals. Briefly, six pairwise comparisons (CF vs. FM, CF vs. click bat, CF vs. toothed whale, FM vs. click bat, FM vs. toothed whale, and click bat vs. toothed whale) were conducted to detect parallel/convergent sites between the two members of each pairwise comparison. The parallel/convergent sites among the pairwise comparisons of each gene were identified in accordance with previously described methods (Foote *et al.* 2015). Then, we used the software CONVERG 2 (Zhang and Kumar 1997) to test whether the observed convergent/parallel substitutions in focal branches had been fixed randomly or were due to natural selection.

### Localization of important sites

Amino acid substitutions in key protein functional domains or transmembrane regions may affect the physicochemical properties and functions of a specific protein. *SK2* is an important gene coding an ion channel protein; positively selected sites in the sequence may reflect potential changes of functional properties. The protein domains and transmembrane topology of SK2 were predicted and plotted according to InterProScan (http://www.uniprot.org/) and TMHMM Server v. 2.0 (http://www.cbs.dtu.dk/services/TMHMM/). Subsequently, we mapped all the positively selected sites onto the schematic plot of the SK2 protein to illustrate its potential changes in echolocating mammals.

## Results

### Phylogenetic reconstruction

**Figure 3.**
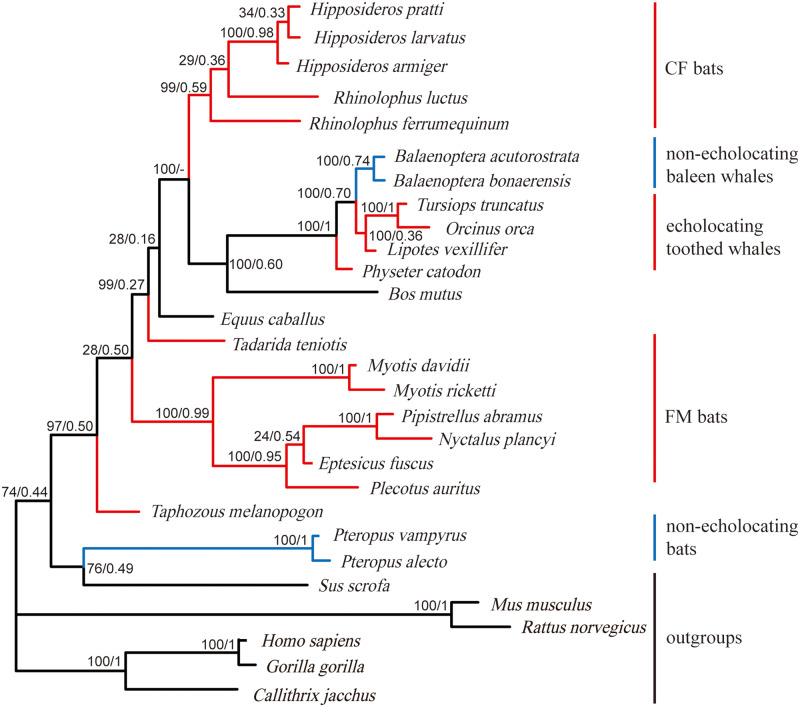
Gene trees based on *Shh* coding region sequences for 29 mammals. Values on the branches indicate statistical support from ML and BI analyses. The negative sign (−) indicates a lack of statistical support for a specific method. Branch colors are in accordance with Figure 1.

In total, 246 bp coding region sequences of the *Shh* gene for 29 mammals were successfully sequenced and collected (Table 1). Among which, 11 newly sequenced bat species are listed in bold in Table 1. Phylogenetic trees of the *Shh* gene based on ML and BI methods showed similar topological structures (Figure 3). In particular, all echolocating bats and whales and several other mammals were “erroneously” grouped together with strong support (97% ML support and 0.5 Bayesian posterior probability). CF bats and whales were grouped together with 100% ML support, and then these were grouped together with FM bats. Two nonecholocating bat species, *P. vampyrus* and *P. alecto*, however, formed a separate clade with pig, and these were grouped together with primate and rodent species that produced a tree topology different from previously reported mammalian species trees (Figure 1). However, there were no parallel/convergent sites on the *Shh* gene between echolocating bats and whales or in any other pairwise comparisons of echolocating mammals.

**Figure 4.**
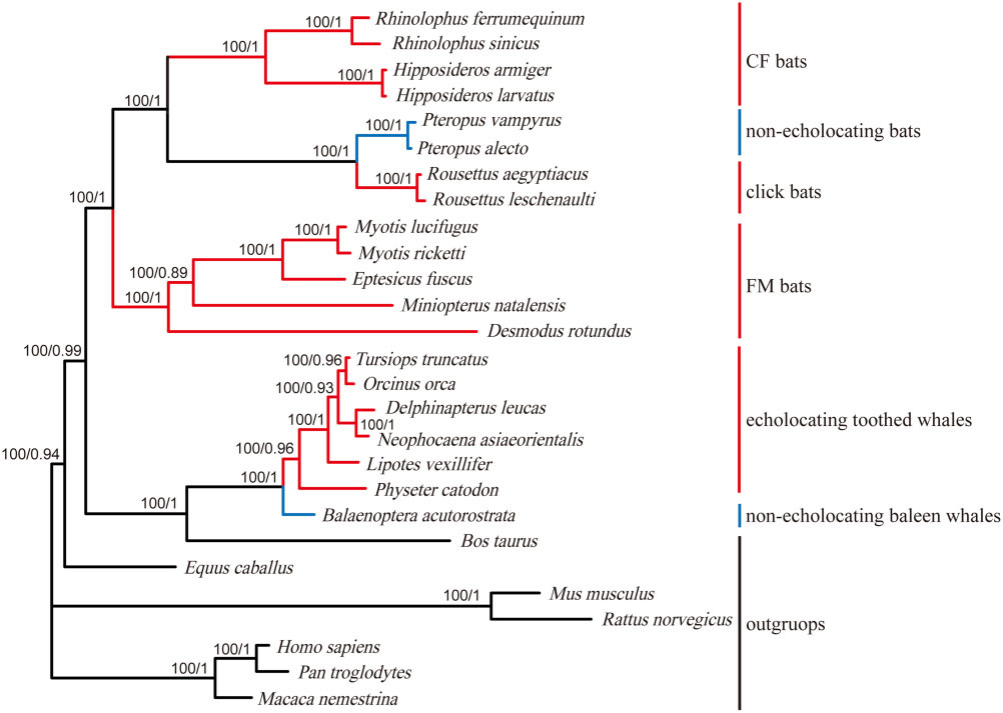
Gene trees based on *SK2* coding region sequences for 27 mammals. Values on the branches indicate statistical support from ML and BI analyses. The negative sign (−) indicates a lack of statistical support for a specific method. Branch colors are in accordance with Figure 2.

We sequenced and aligned 2,712 bp of the *SK2* gene for coding region sequences from 27 mammals. Five new sequences from bat species are listed in bold in Table 2. Phylogenetic trees of the *SK2* gene based on ML and BI methods showed similar topologies (Figure 4) with the real species tree (Figure 2). No parallel/convergent sites were found for the *SK2* gene in any pairwise comparisons of echolocating mammals.

### Molecular evolution analyses for the *Shh* gene

For the positive selection tests by PAML, only results based on the species trees are demonstrated and discussed here for both *Shh* and *SK2* (Tables 3 and 4 and Supplementary Tables S2–S6), since similar results were obtained when these analyses were repeated using the putative gene topologies (Supplementary Tables S7–S12 and Supplementary Figures S1–S2).

**Table 3:** Results of Branch model analyses for the *SK2* gene based on the species tree. Foreground branches are shown as in Figure 2

Foreground branch	Parameter estimates	LnL	2ΔLnL	d.f.	**Adjusted** ***P*-value**
M0 (one-ratio)	ω = 0.05440	−6836.33	—	—	—
Free-ratio	—	−6807.80	—	—	—
Branch model (two-ratio)		—	—	—	—
Branch a	ω_0_ = 0.05465, ω_1_ = 0.04528	−6836.30	—	—	—
Branch b	ω_0_ = 0.05394, ω_1_ = 0.06720	−6836.26	—	—	—
Branch c	ω_0_ = 0.05502, ω_1_ = 0.03616	−6836.17	—	—	—
Branch d	ω_0_ = 0.05490, ω_1_ = 0.02978	−6836.14	—	—	—
Branch e	ω_0_ = 0.05545, ω_1_ = 0.00010	−6835.33	—	—	—
Branch f	ω_0_ = 0.05393, ω_1_ = 0.25985	−6835.81	—	—	—
Branch model (two-ratio, ω_1_=1)				—	—
Branch a	ω_0_= 0.05441, ω_1_=1.00000	−6849.28	—	—	—
Branch b	ω_0_= 0.05375, ω_1_=1.00000	−6852.16	—	—	—
Branch c	ω_0_= 0.05456, ω_1_=1.00000	−6852.74	—	—	—
Branch d	ω_0_= 0.05476, ω_1_=1.00000	−6847.58	—	—	—
Branch e	ω_0_= 0.05457, ω_1_=1.00000	−6845.61	—	—	—
Branch f	ω_0_= 0.05387, ω_1_=1.00000	−6836.10	—	—	—
LRT of variable ω values among branches		—	—	—	—
Free-ratio vs. M0	—	—	57.06	50	0.23
LRT of ω at specific lineages (two-ratio vs. M0)					
Branch a	—	—	0.06	1	0.81
Branch b	—	—	0.14	1	0.85
Branch c	—	—	0.32	1	0.86
Branch d	—	—	0.38	1	1.00
Branch e	—	—	2.00	1	0.96
Branch f	—	—	1.04	1	0.93
LRT of ω at specific lineages (two-ratio vs. two-ratio, ω_1_=1)					
Branch a	—	—	25.96	1	<0.001
Branch b	—	—	31.80	1	<0.001
Branch c	—	—	33.14	1	<0.001
Branch d	—	—	22.88	1	<0.001
Branch e	—	—	20.56	1	<0.001
Branch f	—	—	0.58	1	0.45

Significant value of *P *<* *0.05 for LRT.

**Table 4:** Results of Site model analyses for the *SK2* gene based on the species tree

Site model	Parameter estimates	LnL	2ΔLnL	d.f.	**Adjusted** ***P*-value**	Nominally significant positively selected sites
M0 (one-ratio)	ω = 0.05440	−6836.33	—	—	—	None
M1a (neutral)	*p*_0_ = 0. 95693 (*p*_1_ = 0. 04307) ω_0_ = 0. 02194, ω_1_ = 1.00000	−6799.53	—	—	—	None
M2a (selection)	*p*_0_ = 0.95693, *p*_1_ = 0.00740 (*p*_2_ = 0.03567) ω_0_ = 0.02194, ω_1_ = 1.00000, ω_2_ = 1.00210	−6799.53	—	—	—	55 P (0.508), 97 A (0.659), 227 L (0.624), 286 A (0.642), 774 A (0.642), 784 A (0.604)
M3 (discrete)	*p*_0_ = 0.95693, *p*_1_ = 0.01372 (*p*_2_ = 0.02935) ω_0_ = 0.02194, ω_1_ = 1.00000, ω_2_ = 1.00010	−6796.24	—	—	—	None
M7 (beta)	*p* = 0.04033, *q* = 0.28402	−6807.83	—	—	—	None
M8 (beta&ω > 1)	*p*_0_ = 0.96828, *p* = 0.04181, *q* = 0.38394, (*p*_1_ = 0.03172), ω = 1.00023	−6797.92	—	—	—	55P (0.622), 93S (0.639), 97 A (0.867), 227 L (0.834), 286 A (0.878), 774 A (0.849), 782H (0.531), 784 A (0.841)
LRT of variable ω values among sites		—	—	—	—	—
M0 vs. M3	—	—	80.18	4	<0.001	—
M1a vs. M2a	—	—	0.00	2	1	—
M7 vs. M8	—	—	19.82	2	<0.001	—

Significant value of *P *<* *0.05 for LRT.

*Note*: Numbers in the bracket following nominally significant positively selected sites are the corresponding posterior probability

For the Site model in PAML (Supplementary Table S2), the LRT result of M0 vs. M3 (2ΔLnL = 36.30, d.f. = 4, adjusted *P *<* *0.001) suggested that the *Shh* gene is not under neutral evolution. However, no positively selected sites were detected based on the results of the Site model by PAML or Datamonkey.

The results for the Branch-site model showed that one site (19 C) with ω  >  1 was detected along the CF bats branch (branch c, Figure 1), and two sites (13 V and 19 C) with ω  >  1 were detected along the Hipposideridae branch (branch b, Figure 1). However, the adjusted *P*-values of LRT were not significant (Supplementary Table S3).

**Figure 5.**
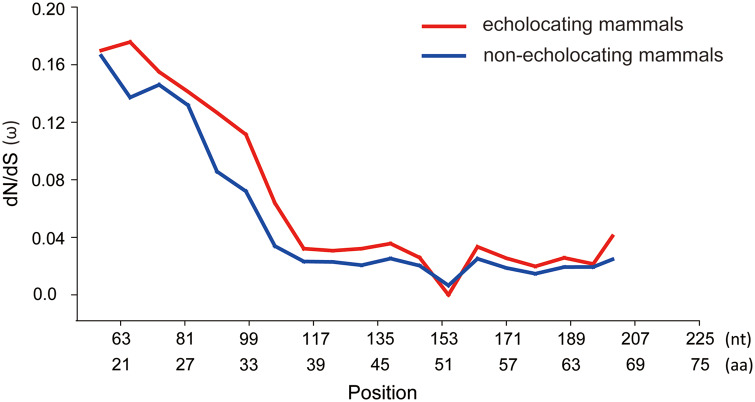
The variation of ω values evaluated along *Shh* genes in 17 echolocating mammals and 12 nonecholocating mammals according to sliding window analysis.

Different results for the Branch model were produced by PAML and Datamonkey. The ω-values detected in echolocating branches (a–e, Figure 1) were not significantly greater than the values detected in the corresponding background branches based on the results from PAML (Supplementary Table S4). However, according to the results using the FEL model in Datamonkey, the ω value of all combined echolocating mammals was significantly greater than the value for the remaining nonecholocating mammals (foreground branch ω  =  0.107, background ω  =  0.0963, adjusted *P *<* *0.001), indicating that various selective pressures were acting on the *Shh* gene, perhaps higher adaptation in echolocating mammals. Similar results were obtained by the sliding window analyses, Figure 5 shows that the estimated ω values in 17 echolocating species were always significantly greater than those detected in 12 nonecholocating species (*P *=* *0.03).

### Molecular evolution analyses for the *SK2* gene

Nominally significant positively selected branches and sites were identified by the Branch model (Table 3) and the Site model (Table 4 and Supplementary Table S5) but not in the Branch-site model (Supplementary Table S6) according to PAML and Datamonkey analyses. First, there were seven branches with ω > 1 identified by the free-ratio model in the Branch model (Figure 2), and these seven branches all led to echolocating species. However, the LRT of free-ratio vs. M0 was not significant (2ΔLnL = 57.06, d.f. = 50, adjusted *P *=* *0.23), indicating that the *SK2* gene was conserved through evolution (Table 3). Second, the ω value of the Hipposideridae branch (Branch b) was significantly greater than those of background branches (adjusted *P *<* *0.001, Table 3) in the Branch model, indicating perhaps stronger selective pressures or lower selective constraints on the *SK2* gene in Hipposideridae species. In addition, the *K*-value was significantly greater than 1 (*K* = 2.03, *P *=* *0.03; *K* is a parameter estimating the intensity of selection used as an index for the correction of ω) when we set all echolocating species as foreground branches in the RELAX model implemented in Datamonkey, suggesting that the *SK2* gene may have undergone stronger selective pressures in echolocating species compared with nonecholocating species. Moreover, the ω-value was 0.0769 in foreground branches when we combined all echolocating species and 0.0738 when we combined laryngeal echolocating bats as foreground branches, both of which were significantly larger than the ω-values from the corresponding background branches (0.0518 and 0.0548, respectively), suggesting different selective pressures acting on the *SK2* gene in echolocating mammals compared with nonecholocating mammals.

Several nominally significant positively selected sites were detected by the multiple Site models (Table 4 and Supplementary Table S5). According to the results from PAML, the LRT of M0 vs. M3 and M7 vs. M8 were significant, and there were eight nominally significant positively selected sites (55 P, 93S, 97 A, 227 L, 286 A, 774 A, 782H, and 784 A) in M8 (Table 4). In addition, several positively selected sites were also detected by Datamonkey, including one site (227) by the MEME model, one site (227) by the FEL model, and one site (286) by the FUBAR model before multiple test correction (Supplementary Table S5). In particular, sites 227 and 286 were detected by both PAML and Datamonkey, indicating possibly important roles in the evolution of the *SK2* gene. However, no positively selected sites were detected by Datamonkey after multiple test correction.

### Distribution of the nominally significant positively selected sites in the SK2 protein structure

**Figure 6.**
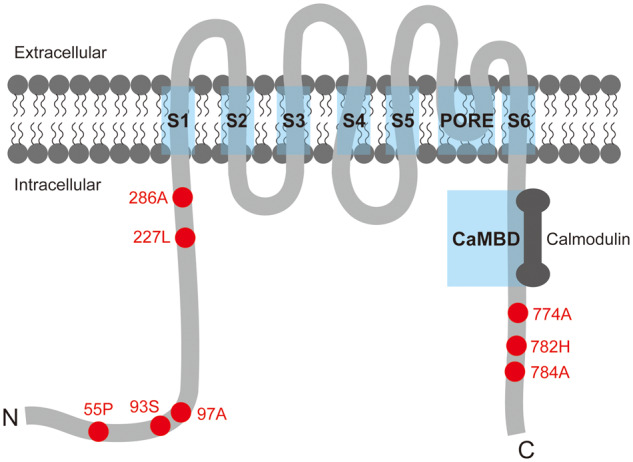
Schematic plot of the SK2 protein structure. Blue rectangles cover the eight functional domains, including transmembrane segments (S1–S6), the pore region (PORE), and the calmodulin binding domain (CaMBD). Red points with labeled numbers indicate nominally significant positively selected sites detected by PAML and Datamonkey.

The SK2 protein structure was plotted according to the predicted results (Figure 6), comprising six transmembrane segments (S1–S6), one pore region between S5 and S6, and one CaMBD region with 80 amino acids (the calmodulin binding domain). Then, we mapped the nominally significant positively selected sites onto the protein structure and found that those sites were located in the N terminus region or the C terminus region rather than in those functional domains.

**Figure 7.**
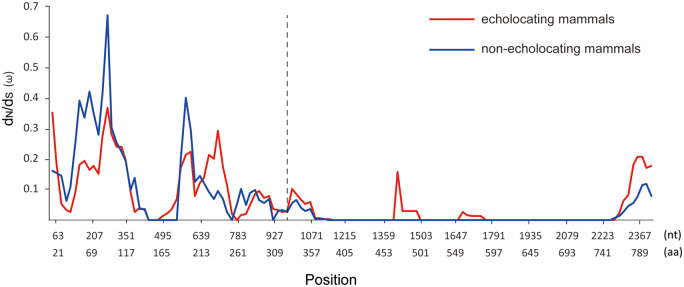
The variation in ω values evaluated along *SK2* genes in 17 echolocating mammals and 10 nonecholocating mammals according to the sliding window analysis.

According to the results of the sliding window analysis, ω values varied greatly among both the 17 echolocating mammals and the 10 nonecholocating mammals in different regions of the *SK2* gene (Figure 7). From the N terminus region to S1, ω values in 17 echolocating mammals and 10 nonecholocating mammals changed frequently, indicating that this part of the *SK2* gene had a high degree of variability in all tested mammals. However, ω values approached zero from S1 to the C terminus region for both echolocating and nonecholocating mammals, and the difference between the two clades of ω values detected in echolocating and nonecholocating mammals was not significant (*P *=* *0.06), suggesting that this part of the *SK2* gene was conserved in the tested mammals. At the same time, the ω-values at several amino acid sites in echolocating mammals were still greater than those in nonecholocating mammals, indicating that those sites experienced different selective pressures in echolocating mammals compared with nonecholocating mammals.

## Discussion

Echolocation evolved independently in bats and toothed whales, and this has been used as an example of “good design” through evolution via natural selection (Dawkins 1986; Au and Simmons 2007). The molecular mechanisms behind the well-developed echolocation in bats and whales have long been an intriguing topic. Previous studies reported several hearing-related genes to be under adaptive evolution during the development of echolocation, including *Prestin* and *TMC1* (Li *et al.* 2008; Davies *et al.* 2012; Shen *et al.* 2012). Here, we identified and analyzed two additional important candidate hearing-related genes, *Shh* and *SK2*, that may have experienced selection during the evolution of echolocation. Comprehensive molecular evolutionary analyses of *Shh* and *SK2* suggested that they may play important roles in the hearing process and may have experienced higher adaptation during the development of high-frequency hearing in echolocating mammals. Therefore, *Shh* and *SK2* are two important new subjects for studies of adaptive evolution in hearing-related genes.

Phylogenetic trees of *Shh* and *SK2* genes showed different relationships among mammals with or without echolocating ability. The reconstructed phylogenetic trees for the *Shh* gene consistently grouped together all echolocating bats and whales and excluded nonecholocating Pteropodidae bats (Figure 2), in contrast to the well-established species trees (Figure 1). Similar results have been reported for several important hearing-related genes such as *Tmc1* and *pjvk* (Davies *et al.* 2012). Previously, the reconstructed gene trees for *Tmc1* and *pjvk* have united echolocating bats and echolocating toothed whales due to parallel/convergent sites existing in those mammals with echolocating ability. However, no parallel/convergent sites were detected in the *Shh* gene between echolocating bats and whales. The discrepancy between the gene tree and species tree for the *Shh* gene may be due to other reasons, such as long-branch attraction (LBA) or the lack of information for short coding sequences for effective reconstruction of the gene tree. Regarding the other important hearing-related gene, the gene tree for *SK2* showed a similar topology with the species tree. Taken together with the results from previous studies, this suggests that parallel/convergent evolution between echolocating bats and whales may have led to the difference between gene and species trees, whereas the clustering of echolocating bats and whales in the gene tree may have been produced by factors other than parallel/convergent evolution (Li *et al.* 2008; Liu *et al.* 2012; Thomas and Hahn 2015; Zou and Zhang 2015). Other factors may also account for this difference; therefore, additional careful analyses of the gene sequences and amino acid sequences are needed for the analysis of adaptive molecular evolution. Nonetheless, it is still an important and necessary preliminary step to reconstruct the phylogenetic trees for candidate genes, thereby providing effective information for subsequent in-depth analyses of molecular evolution (Li *et al.* 2010; Davies *et al.* 2012; Xu *et al.* 2013).

Multiple positive selection and sliding window analyses consistently indicated that both *Shh* and *SK2* were conserved through evolution. Generally, important functional genes are conserved to maintain functional stability. Previous studies of the *Shh* gene demonstrated its conserved role in tonotopic organization of the avian basilar papilla and mammalian cochlea (Son *et al.* 2015). *SK2* gene expression in the cochlea of mammals is functionally important for normal hearing (Allen *et al.*, 2007, 2011). In addition to being evolutionarily conserved, both *Shh* and *SK2* showed more heterogeneous selection pressures in echolocating mammals than in nonecholocating mammals. Echolocating mammals, such as echolocating bats and toothed whales, have developed high-frequency hearing ability; hence, those hearing-related genes might have experienced higher adaptation to this particular phenotype. Previous studies demonstrated that several hearing-related genes including *Prestin*, *Tmc1*, and *KCNQ4* evolved faster and were positively selected in echolocating mammals compared to nonecholocating mammals (Li *et al.* 2008; Davies *et al.* 2012; Liu *et al.* 2012; Shen *et al.* 2012). Similarly, our results for *Shh* and *SK2* indicated that different selective pressures may have acted on echolocating mammals compared to nonecholocating mammals. Taken together, the results suggest that hearing-related genes may have worked together to contribute to the adaptive evolution of echolocation in mammals.

In addition, compared with the *Shh* gene, we found relatively stronger evidence of adaptive molecular evolution in the *SK2* gene in echolocating mammals, suggesting that various selective pressures may act on different hearing-related genes. In addition, selective pressures were also varied in different areas of this hearing-related gene. Our results based on the adaptive molecular analyses showed that *SK2* was likely to have stronger structural constraints, as those nominally significant positively selected sites were located in the unstructured domain, whereas the functional domain of the SK2 protein was highly conserved. At the same time, similar results were obtained by the sliding window analyses, indicating that different intensities of selective pressure may act on different parts of this gene: functional domains of *SK2* were relatively conserved through evolution, while noncoding functional domains exhibited more rapid evolutionary rates. Our results demonstrated that different parts of the *SK2* gene may be affected by different intensities of selective pressure due to different functions, thus presenting different patterns of evolution during high-frequency hearing development.

Our findings from *Shh* and *SK2* and comparisons with *Prestin* and other genes strongly implicate multiple loci in the acquisition of echolocation in mammals (Li *et al.* 2010; Davies *et al.* 2012). Recently, comparative genomic analyses have been used to unveil the genetic bases underlying adaptive evolution of echolocation in mammals (Liu *et al.* 2018; Wang *et al.* 2020). A number of candidate genes responsible for echolocation and high-frequency hearing were identified; however, these warrant careful analysis in the future. Furthermore, the combination of studies on both the genomic and single gene scales could be more efficient for uncovering the adaptive evolution of echolocation.

In conclusion, two important candidate hearing-related genes, *Shh* and *SK2*, were analyzed and shown to experience faster rates of evolution in echolocating mammals than in nonecholocating mammals. In the *SK2* gene, several nominally significant positively selected sites were detected in the nonfunctional domains by multiple methods, suggesting that the gene may play an important role in the high-frequency hearing of echolocating mammals. The discovery of these two genes could be an important complementary finding to previous studies of adaptive evolution of hearing-related genes. Our study offers important candidate genes and research ideas for future correlational studies. Echolocation is an intriguing topic, and more data and molecular evidence from multiple aspects are needed to uncover the adaptive evolution of echolocation.

## Data availability

The authors affirm that all data necessary for confirming the conclusions of the article are present within the article, figures, and tables. New sequences generated in this article have been deposited into the NCBI (KX495649–KX495659 and MT822706–MT822710). Supplemental material available at figshare: https://doi.org/10.25387/g3.13564214.
